# Transition Metal-Mediated Annulation Approaches for Synthesis of Arylnaphthalene Lignan Lactones

**DOI:** 10.3389/fchem.2020.00628

**Published:** 2020-08-06

**Authors:** Sooyoung Park, Jin-Hee Kim, Seok-Ho Kim, Dongyun Shin

**Affiliations:** ^1^College of Pharmacy, Gachon University, Incheon, South Korea; ^2^College of Pharmacy, Yonsei University, Incheon, South Korea; ^3^Department of Pharmacy, College of Pharmacy and Institute of Pharmaceutical Sciences, CHA University, Pocheon-si, South Korea

**Keywords:** transition metal, arylnaphthalene, catalysis, lignan, synthesis, lactone

## Abstract

Arylnaphthalene lignan lactones belong to a class of natural lignans, and more than 60 analogs have been isolated. Their pharmacological activities as well as unique structural features have attracted considerable attention from medicinal and synthetic chemists. Since the first synthesis in 1895, many synthetic methodologies with ionic or pericyclic reaction mechanisms have been reported. Transition metal catalysts sometimes provide exceptional synthetic versatility for the syntheses of natural compounds. Recently, transition metal-mediated methodologies were investigated for the construction of basic scaffolds of arylnaphthalene lignan lactones. Five kinds of transition metal catalysts containing gold, manganese, nickel, palladium, and silver have been explored. Most of the metal catalysts successfully created arylnaphthalene lactones by intermolecular or intramolecular annulative cyclization. In this review, all reports of transition metal-mediated annulative construction of arylnaphthalene lignan lactones were compiled, and synthetic approaches, mechanistic aspects, and successful applications were discussed.

## Introduction

**Figure 1:**
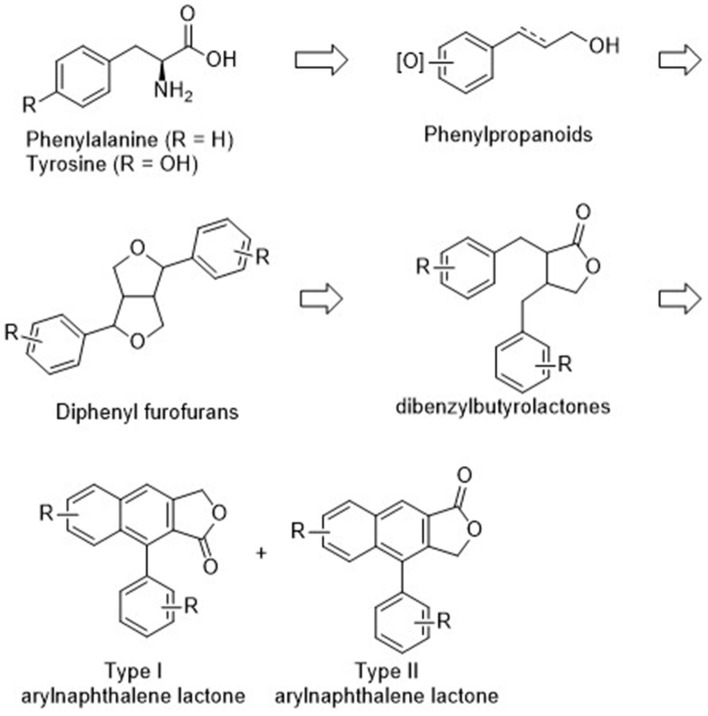
Proposed biosynthetic pathway for arylnaphthalene lignan lactone.

**Figure 2:**
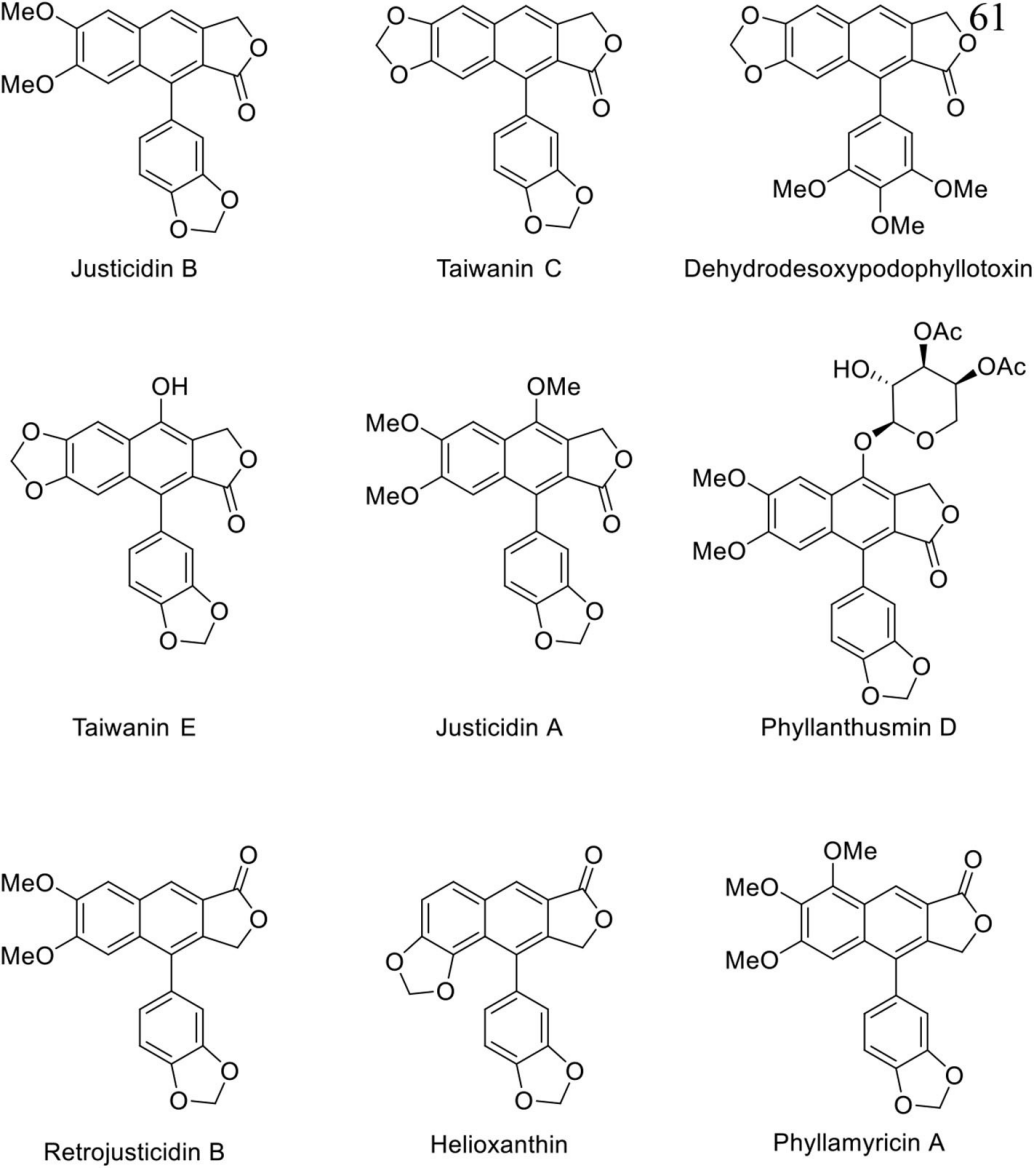
Selected natural arylnaphthalene lignan lactones.

Natural arylnaphthalene lignan lactones are classified as lignans and isolated from a variety of dietary or medicinal plants, such as *Phyllanthus, Justicia, Hapllophyllum*, and *Cleistanthus* (Anjaneyulu et al., 1981; Batsuren et al., 1981; Khalid and Waterman, 1981; Ulubelen, 1985; Lin et al., 1995). They show broad pharmacological activities, including cytotoxic (Day et al., 2002; Yu et al., 2010; He et al., 2012; Deng et al., 2014; Luo et al., 2014; Ren et al., 2014, 2019; Won et al., 2015; Woo et al., 2017; Woodard et al., 2018; Yi et al., 2018; Young et al., 2018), antiplatelet (Chen et al., 1996; Weng et al., 2004), neuroprotective (Gu et al., 2016), antiviral (Asano et al., 1996; Cow et al., 2000), antifungal (Windayani et al., 2014), and anti-HIV (Chang et al., 1995; Zhang et al., 2017) activities. From the biosynthetic viewpoint, oxygenated phenylpropanoids, which are synthesized from phenylalanine or tyrosine, are condensed to bicyclic diphenyl furofurans. Reductive ring opening and oxidative cyclization delivers dibenzylbutyrolactones and final intramolecular Friedel–Crafts type cyclization produced arylnaphthalene lignan lactones (Teponno et al., 2016). Structurally, these compounds can be divided into Type I and Type II arylnaphthalene lignan lactones based on the position of lactones and phenyl groups (Figure 1). Thus far, more than 60 arylnaphthalene congeners and glycosylated products are reported. Diverse and significant pharmacological activities make them more attractive, and representative natural examples are listed in Figure 2.

Since the report of early efforts on the synthesis of arylnaphthalene lignan lactone in 1895 by Michael and Bucher (1895) and in 1910 by Bucher (1910) using the thermal cyclative condensation reaction of arylpropiolic acids, many synthetic methodologies for arylnaphthalene lignan lactones and their applications for natural arylnaphthalene lignan lactones have been investigated. Representative synthetic methodologies include intramolecular Diels–Alder type ring formation of arylpropiolic anhydride by Stevenson's group (Brown and Stevenson, 1964, 1965; Maclean and Stevenson, 1966; Holmes and Stevenson, 1970, 1971; Block and Stevenson, 1971, 1973; Stevenson and Block, 1971; Stevenson and Holmes, 1971; Stevenson and Weber, 1989, 1991; Anastas and Stevenson, 1991; Park et al., 2014), intermolecular Diels–Alder reaction of isobenzofurans and dimethyl acetylenedicarboxylate (De Silva et al., 1980; Plaumann et al., 1980), sequential Blaise reaction-intramolecular [4 + 2] reaction of 2-alkynylbenzonitriles (He et al., 2014), and Garratt–Braverman cyclization of substituted bis-propargyl ethers (Mondal et al., 2011, 2012). Photo-assisted cyclization methods provide arylnaphthalene lignan lactones efficiently (Block and Stevenson, 1971, 1973; Arnold et al., 1973; Yamamoto et al., 2015). Tandem conjugate addition-aldol reaction protocol (Ogiku et al., 1990; Kamal et al., 1994), benzoin condensation-thermal cyclization (Hayat et al., 2015), and electrophilic aromatic substitution protocols (González et al., 1978; Ogiku et al., 1995) have also been reported. Recently, synthetic approaches and biological activities of arylnaphthalene lignan lactones were reviewed by Zhao et al. (2018).

Transition metals have proven to be very useful in modern organic synthesis. The transition metal catalyzed reaction is particularly important for the formation of carbon–carbon bonds, and enables formation of bonds that are not possible with conventional methods. It also allows the formation of complex structures in a single reaction. In this review, all the research articles in which transition metals were utilized for the synthesis of arylnaphthalene lignan lactones are comprehensively discussed and the main advantages in each method are elucidated. There are five transition metals involved in the synthesis of arylnaphthalene lignan lactones, and each one is discussed in terms of the reaction mechanism and its applications for synthesis of arylnaphthalene lignan lactones.

## Transition Metal-Mediated Synthesis of Arylnaphthalene Lignan Lactones

### Gold-Catalyzed Cyclization

**Scheme 1:**
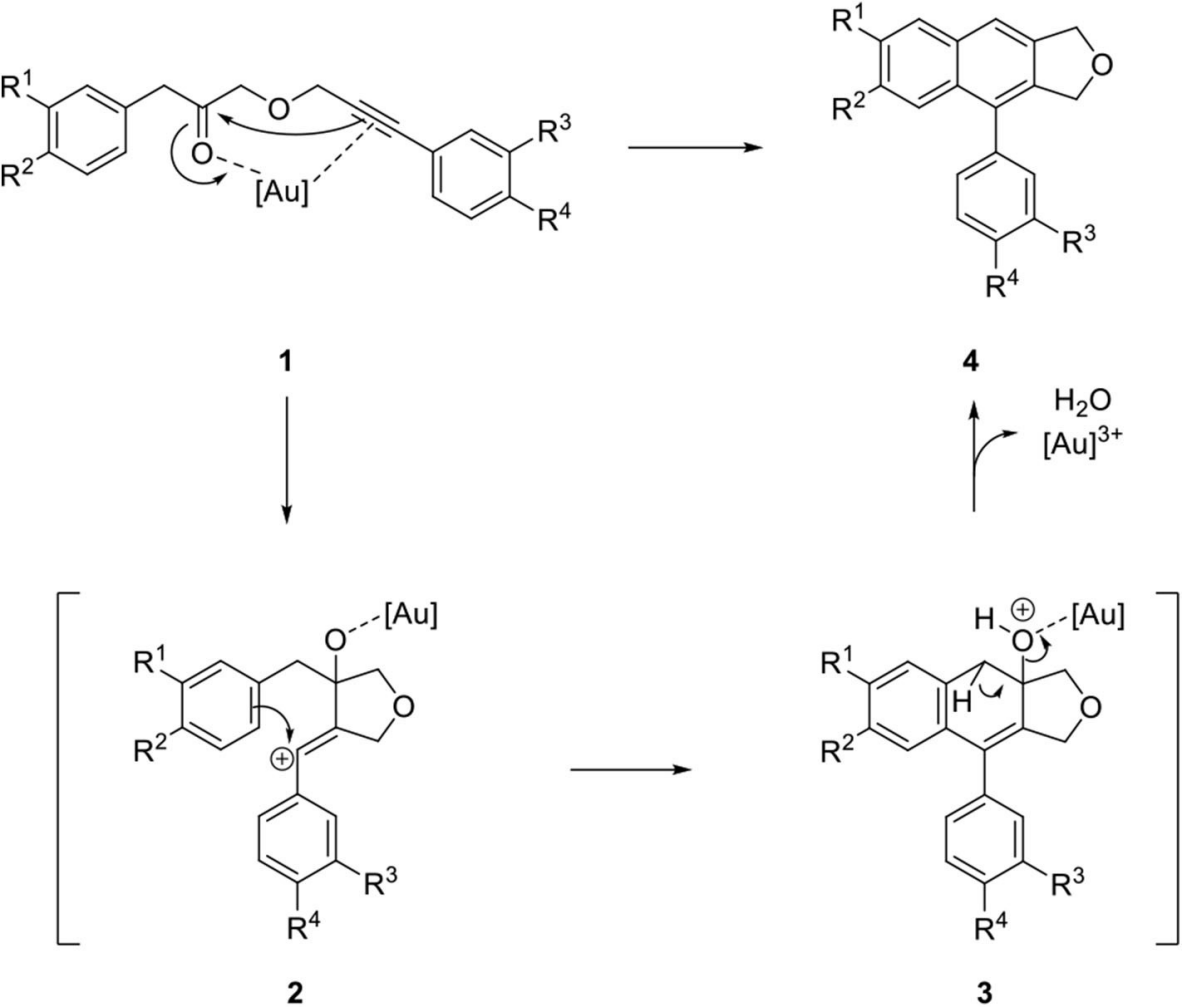
Mechanism of Au-catalyzed intramolecular cyclization of alkynyl ester.

Recently, extensive studies have been devoted to gold-catalyzed reactions in organic synthesis (Hashmi, 2007; Li et al., 2008). Balamurugan's group focused on the intramolecular cyclization of alkynyl ester **1** by an Au catalyst in combination with AgSbF_6_ for the synthesis of an arylnaphthalene lignan lactone lignan scaffold (Gudla and Balamurugan, 2011). Catalysis of the cyclization begins with coordination of the distant carbonyl and alkyne group of **1** to a proximal position by the Au catalyst. Vinyl cation **2** was produced by subsequent attachment of the alkyne on the carbonyl group. Arylnaphthalene lignan lactone precursor **4** was synthesized by the removal of water and Au catalyst from **3** that was obtained from the electrophilic benzannulation reaction of **2** (Scheme 1).

**Scheme 2:**
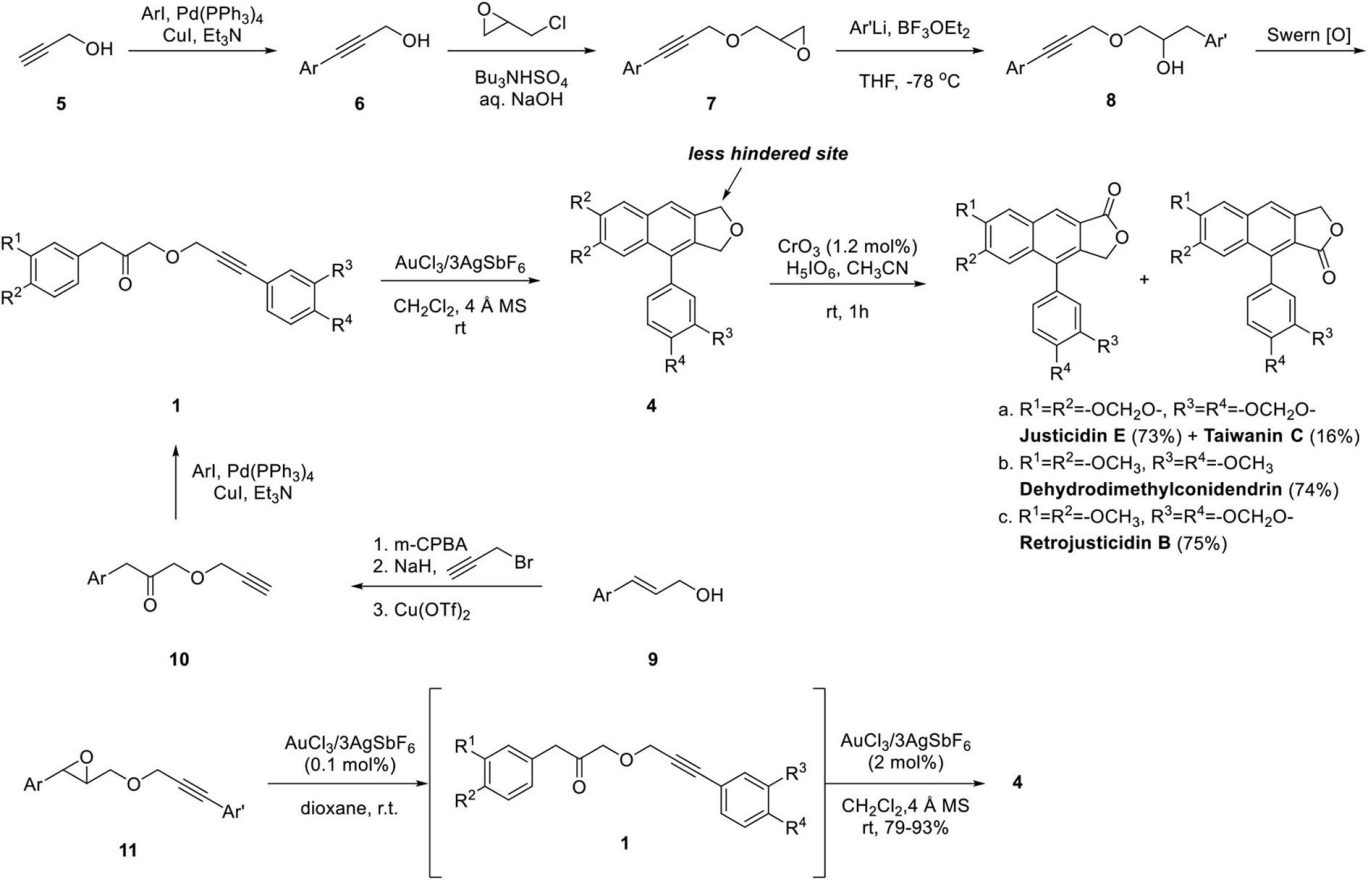
Synthesis of arylnaphthalene lignan lactone by Balamurugan's group.

Application of this methodology to the synthesis of natural arylnaphthalene lignan lactone is summarized in Scheme 2. The key intermediate **1** was obtained from propargyl alcohol in four steps. Sonogashira coupling of propyn-1-ol **5** with substituted aryl iodide provided **6** and **7** was obtained by the substitution reaction. Boron trifluoride-mediated epoxide opening provided **8** and **1** was obtained by Swern oxidation. The ketone **1** can also be obtained by epoxidation of cinnamyl alcohol **9**, propargylation, Cu(OTf)_2_-mediated Meinwald rearrangement and Sonogashira coupling with substituted aryl iodide albeit in low yield. The Au-catalyzed cyclization of **1a**-**1c** was achieved in yields of 84, 68, and 73%, respectively. Regiocontrolled oxidation of **4** by CrO_3_/H_5_IO_6_ in acetonitrile (Yamazaki, 1999) afforded 4.5:1 mixture of type II and isomeric type I arylnaphthalene lignan lactone. The advantage of this synthetic approach is the highly mild reaction condition performed at room temperature and easy derivatization with late-stage introduction of the aryl group from intermediate **10**. Two years later, Balamurugan's group developed the one-pot sequential Meinwald rearrangement of **11** and cyclization of ketone **1** to provide **4** in yields of 79–93% (Gudla and Balamurugan, 2013).

### Manganese-Catalyzed Cyclization

**Scheme 3:**
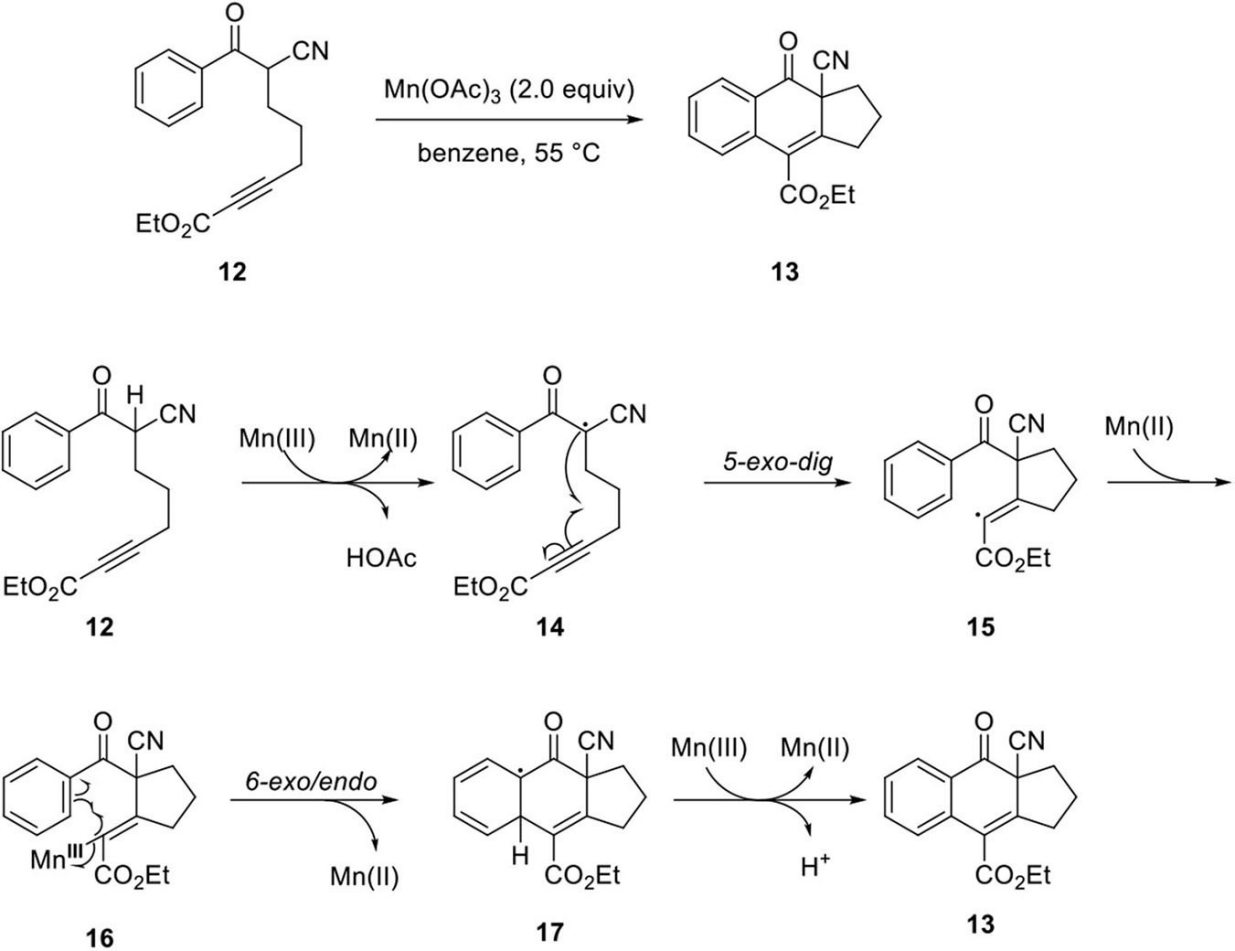
Mechanism of Mn-mediated cyclization.

Shia's group studied Mn(OAc)_3_-mediated cyclization to synthesize arylnaphthalene lignan lactone (Wong et al., 2014; Kao et al., 2015). Initially, they investigated the feasibility of the oxidative cyclization of model system **12**. The reaction proceeded under mild condition compared to the dehydro-Diels–Alder reaction, which requires high reaction temperatures of 160–300°C. The mild reaction conditions allow diverse functional groups, including Trimethylsilyl (TMS), ester, ketone, amide, phosphonate, and halogen. The reaction mechanism was proposed as follows. Abstraction of α-H in **12** produced Mn(III) enolate, and electron transfer generated Mn(II) and radical species **14**. Then, the radical was added to the alkyne by 5-*exo*-*dig* type cyclization to provide **15**. Next, the vinyl radical reacted with Mn(II) to form Mn(III)-vinyl complex **16**, and naphthalene core **13** can be constructed by vinyl radical addition to benzene by 6-*exo*/*endo* type cyclization, and another electron transfer from **17** to Mn(III) ultimately generates an aromatic ring with loss of H^+^ (Scheme 3).

**Scheme 4:**
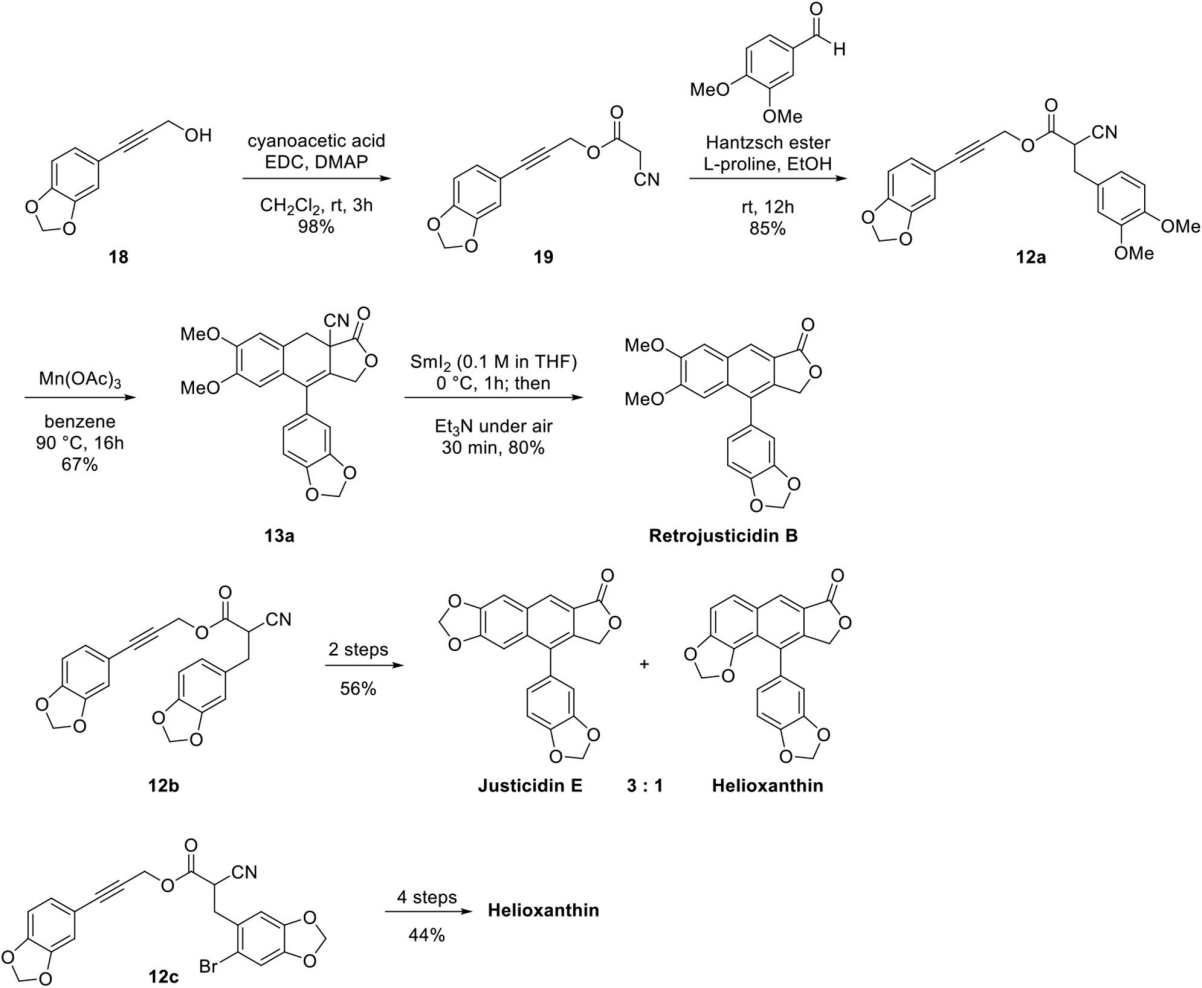
Synthesis of arylnaphthalene lignan lactone by Mn-mediated cyclization.

With these successful results in hand, they turned their attention to the natural products synthesis as shown in Scheme 4. Cyclization precursor **12a** was synthesized by esterification of substituted phenyl propargyl alcohol **18** with cyanoacetic acid, one-pot Knoevenagel condensation with benzaldehyde, and Hantzsch ester mediated reduction. Mn(OAc)_3_-catalyzed cyclization provided inseparable 3:1 mixtures of **13a** and its regioisomer (67:22%). Samarium iodide mediated reductive decyanation and air oxidation afforded retrojusticidin B at 80% yield. Synthesis of justicidin E and helioxanthin was also achieved from **13b** following the same procedure affording a 59:20% mixture. Regioselective synthesis of helioxanthin was accomplished from **13c** by introducing Br in the 2-position of piperonal to minimize regioisomer formation. The main advantage of this reaction may be functional group tolerance; however, the regioselectivity is not satisfactory unless Br is introduced in the 2-position to inhibit adverse cyclization, where Mn(OAc)_3_ is used in stoichiometric amount (2.0 equivalents).

### Nickel-Catalyzed Cyclization

**Scheme 5:**
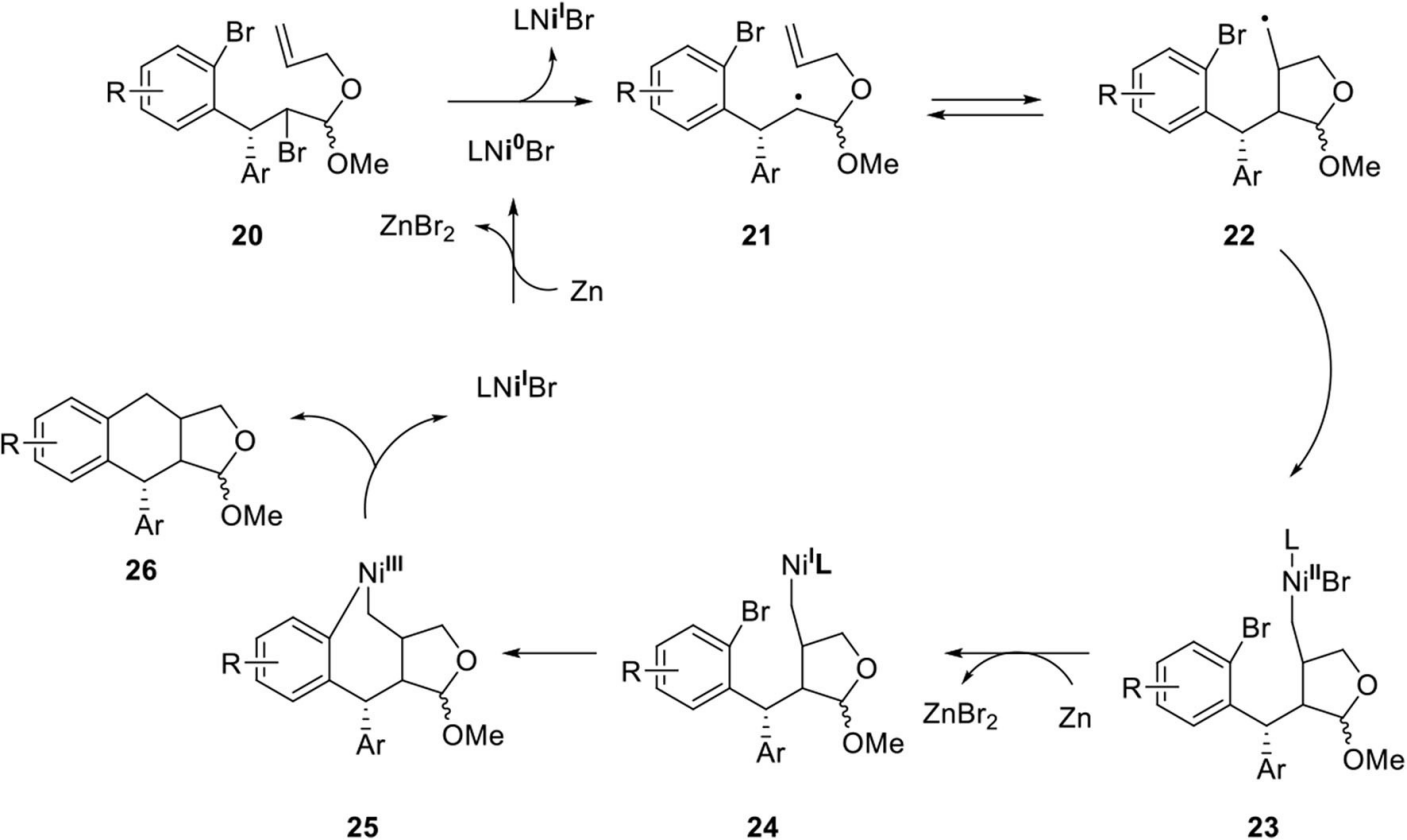
Mechanism of Ni-catalyzed cyclization reaction.

Peng's group developed a nickel-catalyzed cyclization to afford dihydronaphthalene and tetrahydronaphthalene in a diastereodivergent manner (Xiao et al., 2018a). Following on, they investigated the construction of an arylnaphthalene core for the synthesis of dehydrodesoxypodophyllotoxin (Xiao et al., 2018c). Key reaction is summarized in Scheme 5 although detailed stereochemical outcomes are not indicated here (Xiao et al., 2018b). Ni(I) is reduced to Ni(0) by Zinc(0) metal and the single electron transfer process produced alkyl radical **21** from **20**. The secondary radical of **21** was added to the alkene in 5-*exo*-*trig* type producing **22** and then, the primary radical formed the Ni(III) species **2** following the known Ni^I^-Ni^III^ redox process via **23** and **24**. Finally, reductive elimination provided **26** and regenerated the Ni(I) species.

**Scheme 6:**
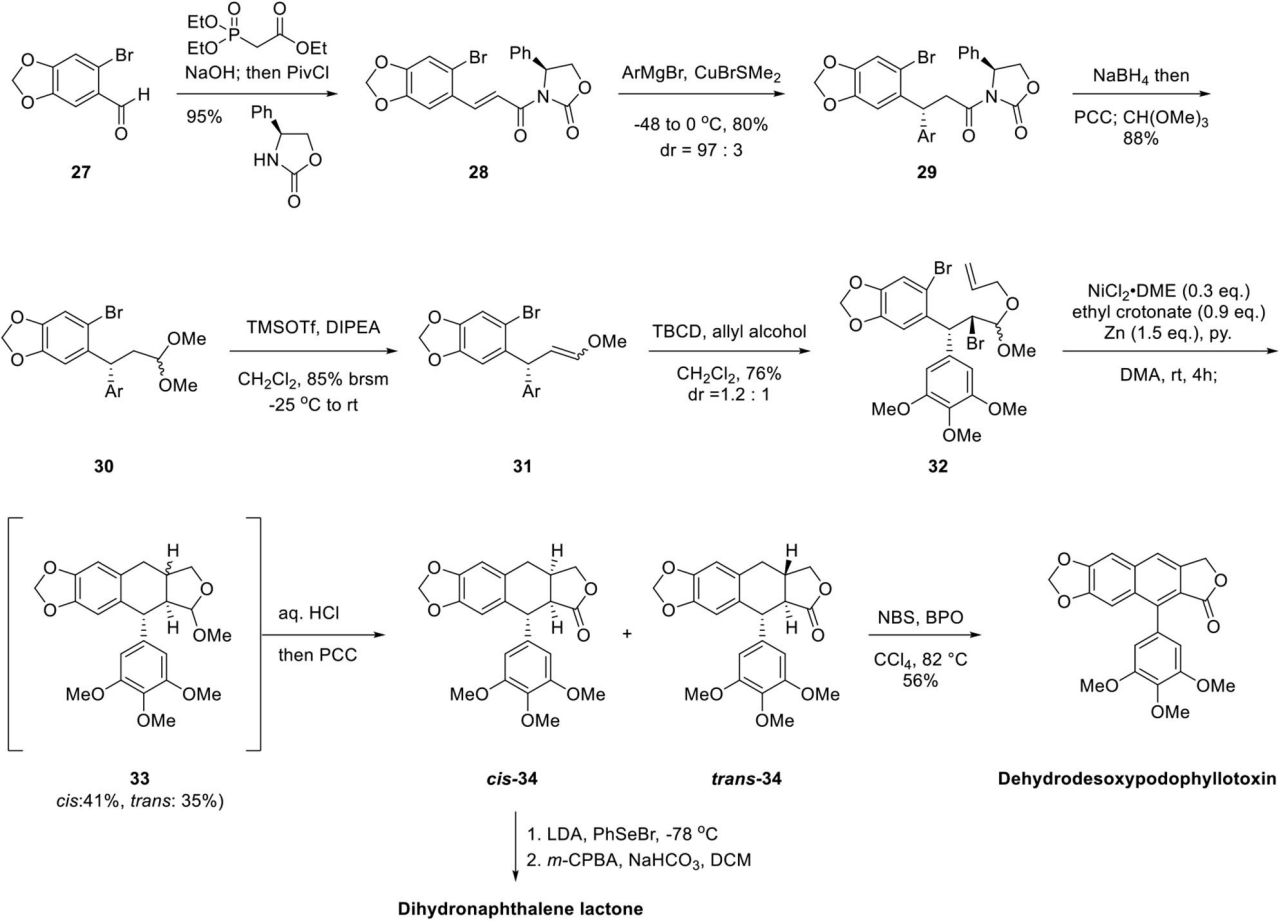
Arylnaphthalene lignan lactone synthesis by Ni-catalyzed cyclization.

The synthesis of arylnaphthalene lignan lactones begins with oxazolidinone imide formation, as shown in Scheme 6. Oxazolidinone imide **28** was obtained by the Horner–Emmons reaction of 6-bromopiperonal **27** with triethyl phosphonoacetate, saponification, and imide formation by reaction of *in situ* generated pivaloyl anhydride and oxazolidinone. Gilman reagent was added to the β-position of enone diasteroselectively at a ratio of 97:3 affording **29** and subsequent reduction of the imide by NaBH_4_, oxidation with Pyridinium ChloroChromate (PCC) and acetal formation afforded **30**. Enol ether **31** was generated in the presence of TMSOTf as Lewis acid. **32** was obtained as a 1.2:1 mixture by bromination with 2,4,4,6-tetrabromo-2,5-cyclohexadienone (TBCD) (Kato et al., 1976, 1984) and concomitant mixed acetal formation with allyl alcohol. Ni-catalyzed reductive cyclization afforded tetrahydronaphthalene lactol ether **33** with *cis* and *trans* mixture of 41 and 35% yield, respectively. Then, acetal **33** was hydrolyzed and oxidized to lactone by PCC to produce ***cis*-34** and ***trans*-34** in 68 and 62% yield, respectively. Dehydrodesoxypodophyllotoxin was obtained by radical halogenation, elimination reaction, and further oxidation from ***trans*-34**. Dihydronaphthalene lactone can be obtained from ***cis*-34** by phenylselenylation at −78°C, *m*-CPBA oxidation, and selenoxide elimination. This Ni-mediated reductive cyclization is advantageous in terms of diastereodivergent synthesis for tetrahydronaphthalene containing diversity-oriented library synthesis.

### Palladium-Mediated Cyclization

**Scheme 7:**
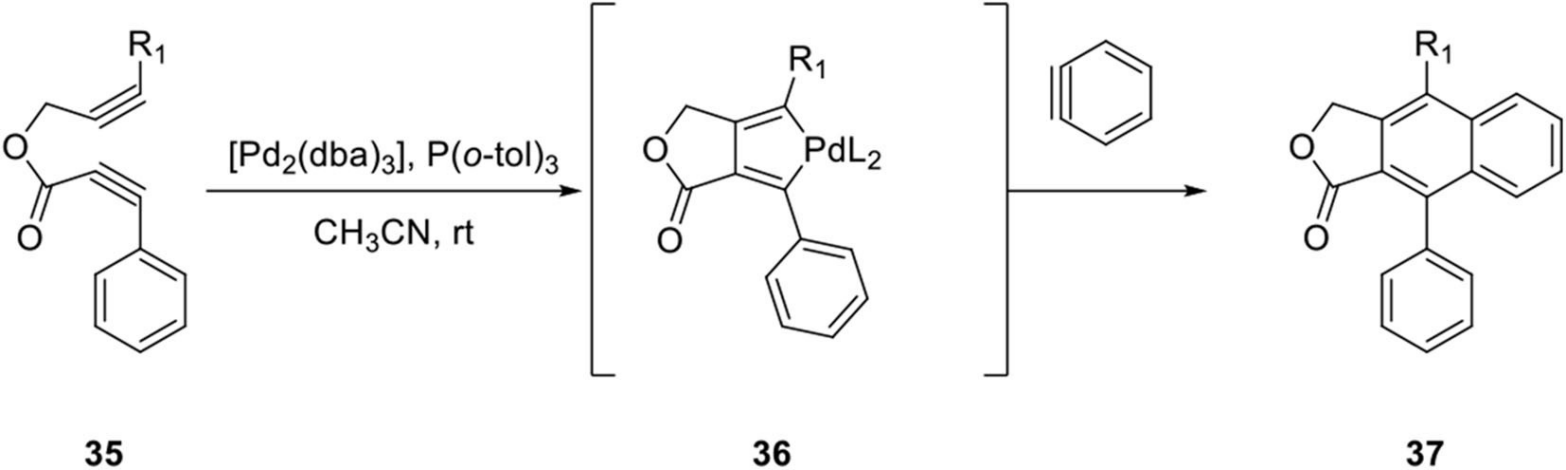
Mechanism of Pd-catalyzed cocyclization of diyne ester and aryne.

Pd-catalyzed copolymerization of diynes and arynes is well-known for the construction of an aromatic ring depending on the metal species (Peña et al., 1998). In continuation of arylnaphthalene lignan synthesis efforts (Mori et al., 1999; Sato et al., 1999), Mori et al. developed a new method for construction of the naphthalene ring by a Pd-catalyzed cocyclization of diyne esters and arynes (Sato et al., 2004). A plausible reaction mechanism can be explained by the formation of palladacycle intermediate **36** from diyne ester **35** and further reaction with benzyne to provide arylnaphthalene lignan lactone **37** (Peña et al., 1998; Scheme 7).

**Scheme 8:**
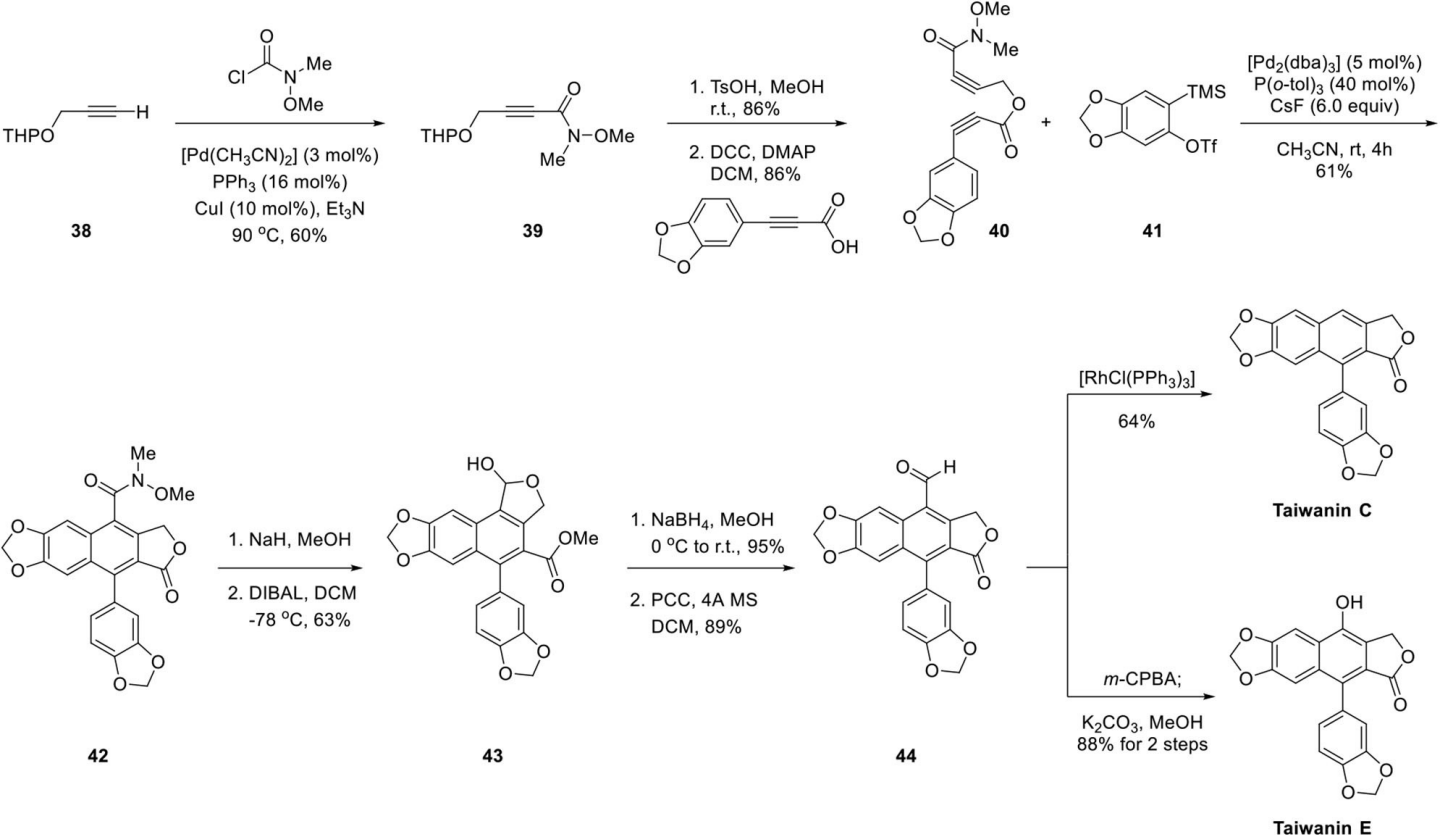
Synthesis of taiwanin C and E by Mori's group.

As depicted in Scheme 8, synthesis of diyne ester **40** was commenced with Pd-catalyzed coupling of *N*-methoxy-*N*-methylcarbamoyl chloride with protected propargyl alcohol **38** (Murakami et al., 1998). After deprotection of **39** in the acidic condition, the resulting alcohol was coupled with 3-[benzo(d)(1,3)dioxol-5-yl]propiolic acid, which was prepared by sequential Corey–Fuchs olefination, carboxylation under *n*-BuLi, and methyl chloroformate, and hydrolysis of methyl ester to afford diyne ester **40**. Aryne precursor **41** was synthesized by silylation of 2-bromophenol, lithiation at low temperature with silyl group migration, and triflation of *in situ* generated phenol. Arylnaphthalene lignan lactone **42** was obtained with diyne ester **40** and aryne precursor **41** in the presence of CsF and Pd catalyst in 61% yield with only 5 mol% loading of Pd catalyst. However, the functional group interconversion of Weinreb amide of **42** was difficult compared to the ease of core ring formation. Direct reduction of amide with LiAl(OtBu)_3_H (Paris et al., 1998) and L-Selectride with MeOTf (Tsay et al., 1990) were attempted but were unsuccessful. Finally, it was converted to aldehyde with an additional four steps. Lactol **43** was obtained by methanolysis of lactone **42** and concomitant transesterification and partial reduction with DIBAL at low temperature. Reduction of lactol **43** with NaBH_4_ afforded diol, which underwent transesterification to provide primary alcohol and aldehyde **44** was obtained upon PCC oxidation. Then, the aldehyde was converted to taiwanin C by the Tsuji–Wilkinson decarbonylation reaction with rhodium catalyst (Tsuji and Ohno, 1965). Taiwanin E was obtained by Baeyer–Villiger oxidation and hydrolysis of the ester.

**Scheme 9:**
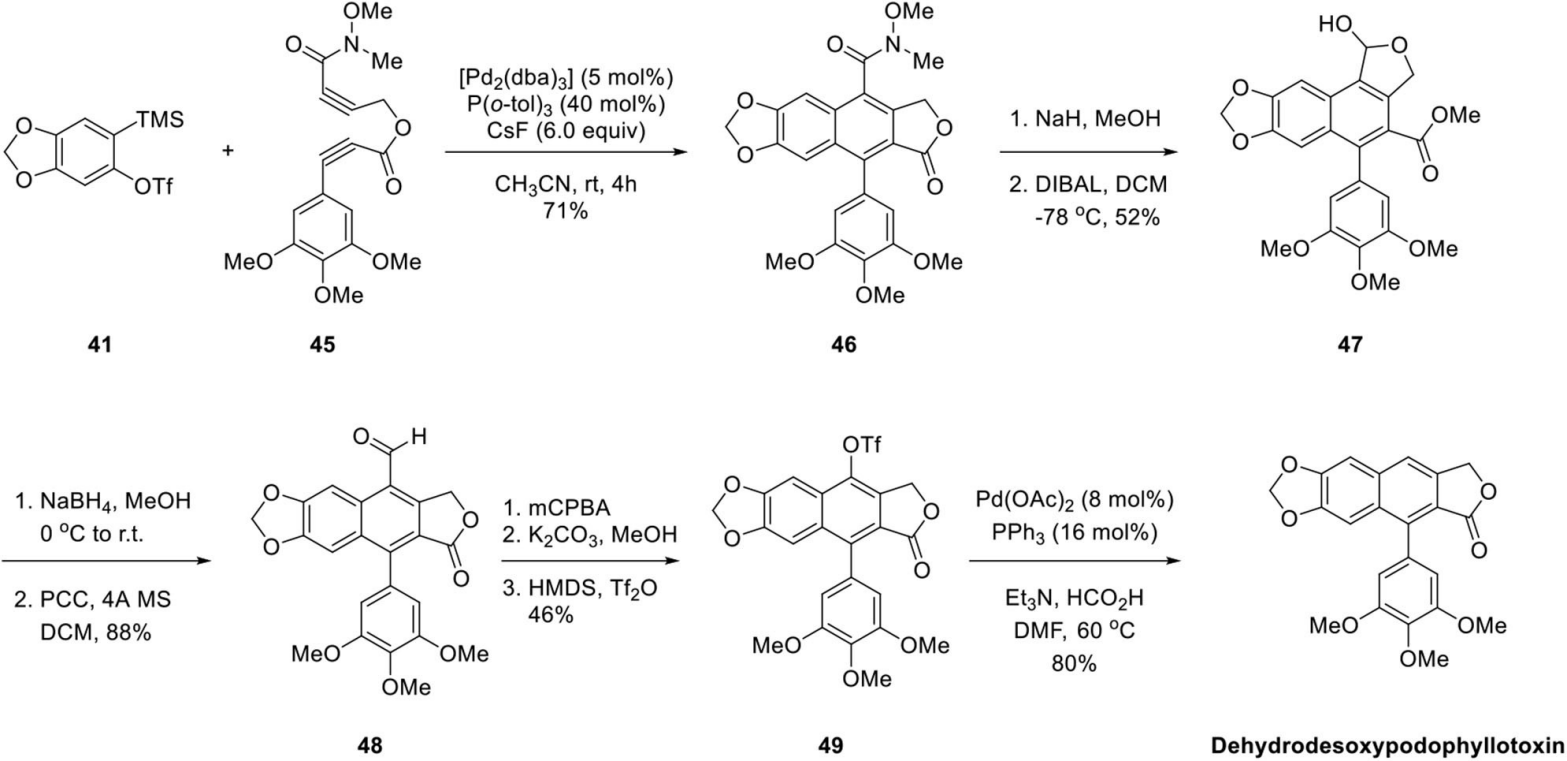
Synthesis of dehydrodesoxypodophyllotoxin by Mori's group.

In 2007, Sato et al. (2007) applied the aforementioned methodology for the synthesis of arylnaphthalene lignan lactone. As shown in Scheme 9, Pd-catalyzed copolymerization of diyne ester **45** and aryne precursor **41** was also successful despite a multistep of reactions for conversion of Weinreb amide **46** to lactol **47** and aldehyde **48**. The aldehyde group of **48** was converted to O-triflate **49** by a three-step sequence of Baeyer–Villiger oxidation, hydrolysis, and triflation of the resulting phenolic hydroxyl group. Finally, the triflate was eliminated by Pd-catalyzed hydrogenation to give arylnaphthalene lignan lactone. The desired dehydrodesoxypodophyllotoxin was synthesized from **46** in low yields of 17% through eight steps. In these two syntheses, constructions of the core ring were highly efficient and regioselective. Further manipulation of the functional groups successfully delivered natural arylnaphthalene lactone products.

**Scheme 10:**
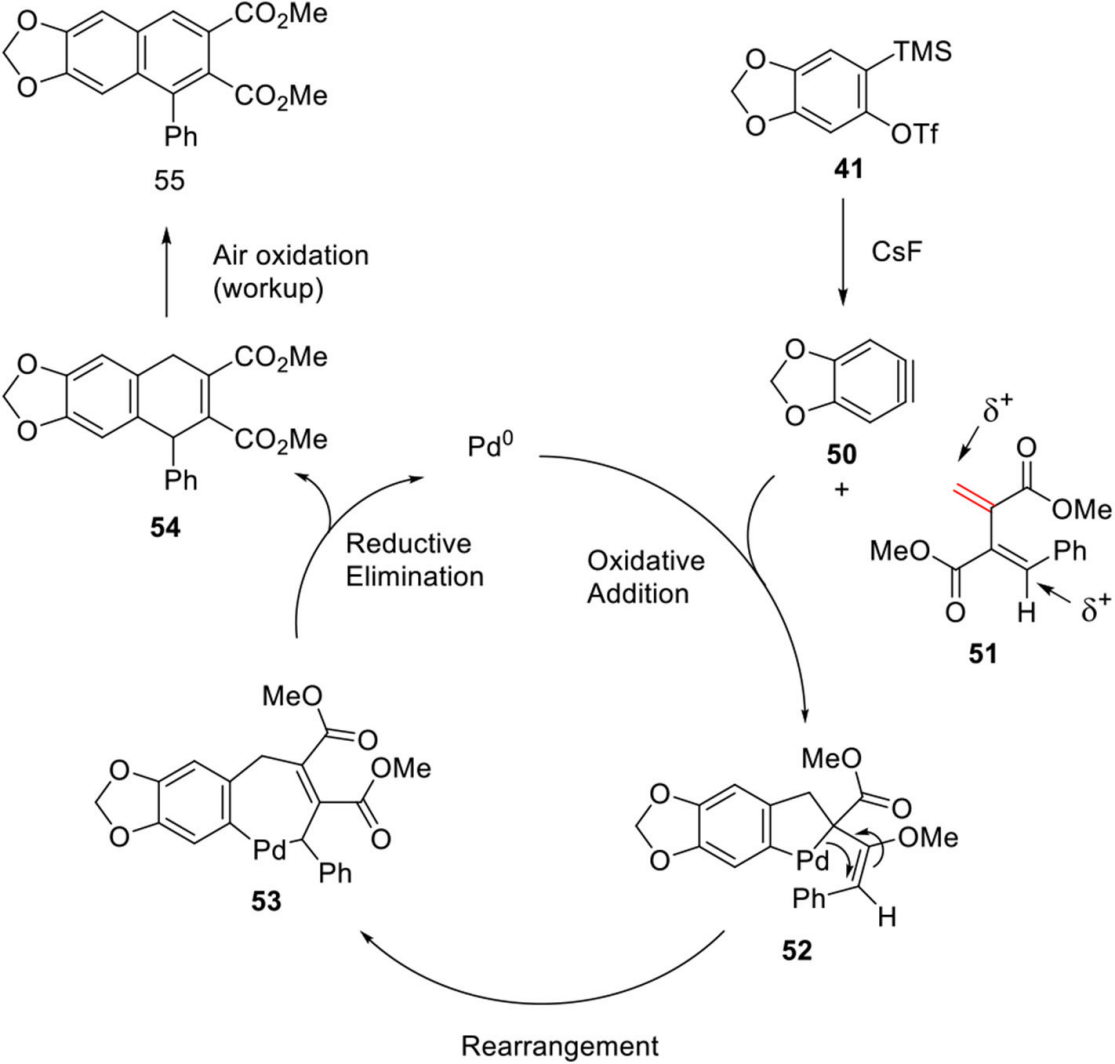
Mechanism of Pd-catalyzed cocyclization of aryne and unsymmetrical diene.

In 2013, Patel and Argade (2013) further developed Pd-catalyzed [2 + 2 + 2] cocyclization of aryne **50** and unsymmetrical conjugated diene **51** using palladium catalyst with *N*-heterocyclic carbene ligand. Initial trial of aryne **50** and diene **51** in the absence of catalyst resulted in only [2 + 2] cycloaddition adduct in which the less substituted olefin of **51** reacted with aryne **50**. It was assumed that the desired [2 + 2 + 2] cycloadduct was not generated because of two electro-positive carbons in diene. Therefore, Argade's group systematically studied metal-mediated [2 + 2 + 2] cocyclization to construct a 6-membered ring. The reaction was ultimately optimized to 62% yield with Pd_2_(dba)_3_ and IMes.HCl as the ligands. The mechanistic pathway involves oxidative addition of Pd to aryne **50** and the less hindered alkene of **51** to form a five-membered palladacycle intermediate **52**, and insertion of more substituted bonds to form a seven-membered palladacycle **53** via rearrangement. Finally, **55** was obtained by reductive elimination of **53** and spontaneous air oxidation of **54** during workup (Scheme 10).

**Scheme 11:**
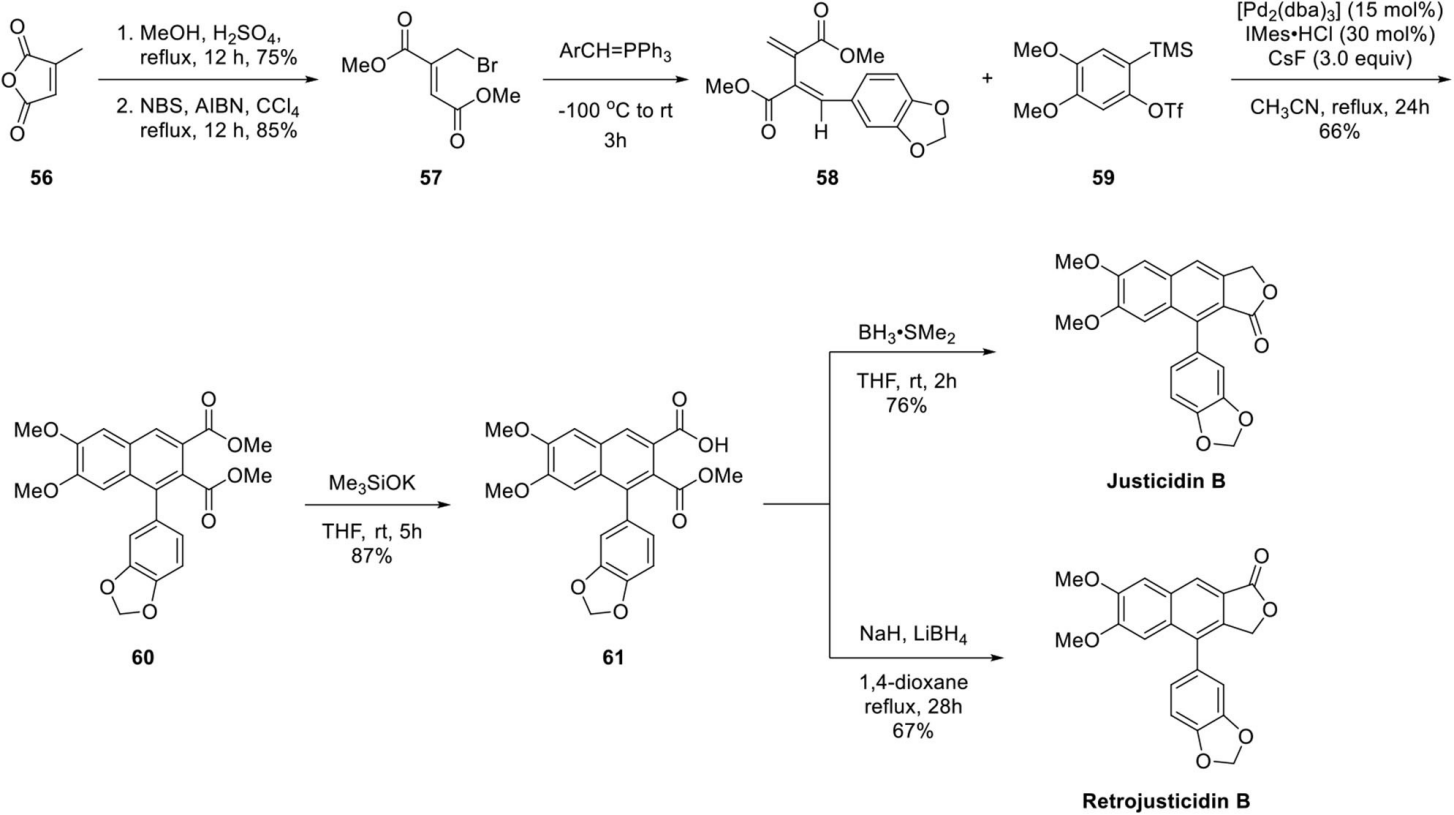
Synthesis of Justicidin B and Retrojusticidin B by Argade's group.

By applying this methodology, the authors successfully achieved total synthesis of Type I and Type II arylnaphthalene lignan lactones, justicidin B and retrojusticidin B, respectively, as presented in Scheme 11. The synthesis begins with methanolysis of citraconic anhydride **56** in acidic condition and then allylic bromination to afford **57** (Kar and Argade, 2002). Unsymmetrical diene **58** was synthesized by further reaction with Wittig reagent (Patel and Argade, 2007). Arylnaphthalene lignan lactone **60** was obtained in 66% yield by Pd-promoted copolymerization of **58** and **59**. Regioselective hydrolysis of less-hindered ester afforded **61**. Justicidin B was obtained by borane reduction of carboxylic acid and lactonization. Retrojusticidin B was obtained in 67% yield by selective ester group reduction in the presence of carboxylate salt with 28% yield of justicidin B as a regioisomer.

**Scheme 12:**
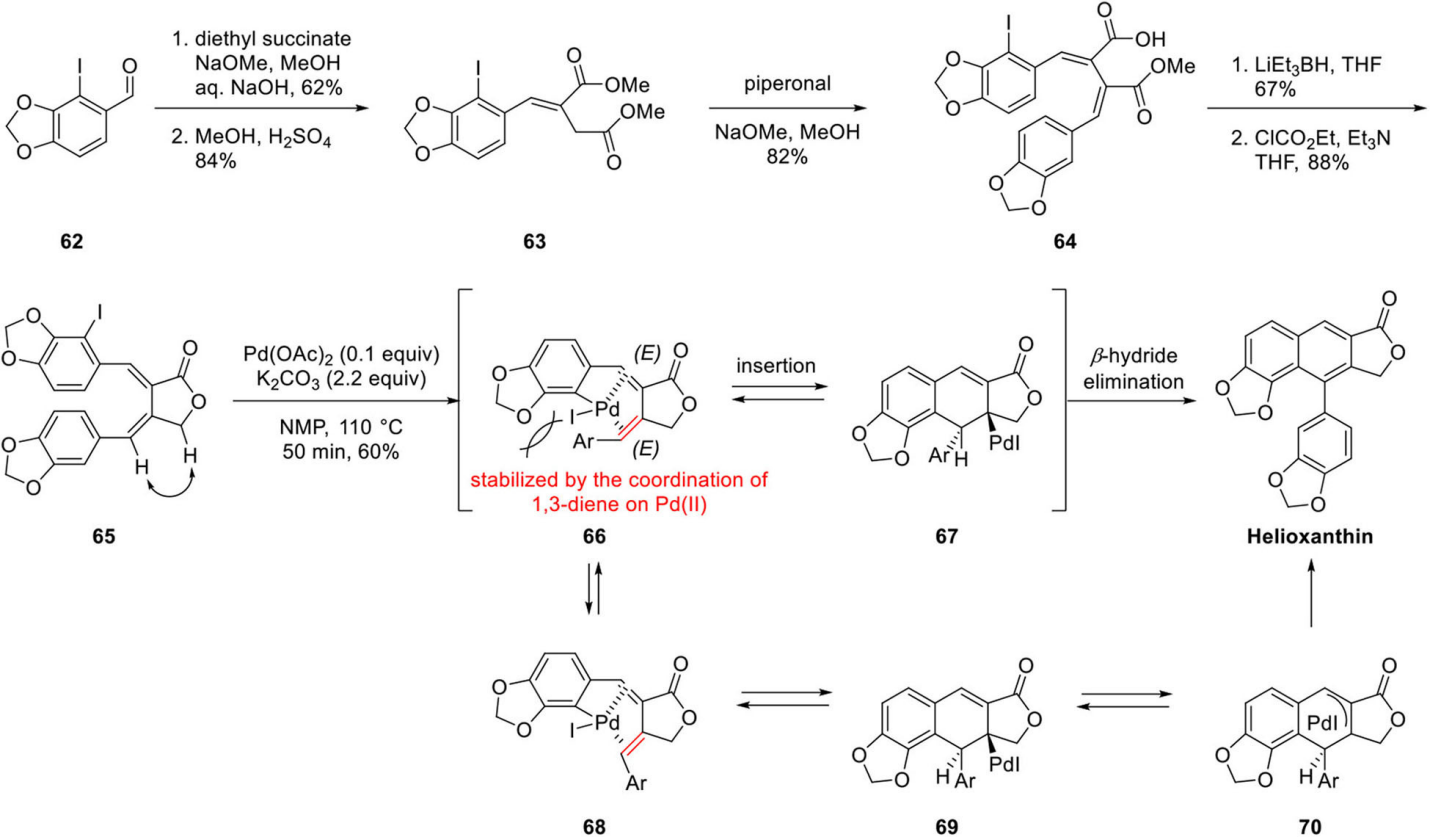
Regiospecific Pd-catalyzed benzannulation by Mitsudera's group.

Mitsudera's group studied the construction of arylnaphthalene lignan lactone by a regiospecific Pd-catalyzed benzannulation of α, β-bisbenzylidene-γ-lactone for the synthesis of helioxanthin (Mizufune et al., 2001; Scheme 12). Structurally, helioxanthin is a 7,8-substituted naphthalene lignan lactone compound, whereas most natural arylnaphthalene lignan lactones bear 6,7-oxygen substituents. Therefore, regioselectivity is the most significant issue in this synthesis. Benzannulation precursor **65** was synthesized by Stobbe condensation of diethyl succinate with 2-iodopiperonal **62** and Fischer esterification to afford **63**. Stobbe reaction of **63** with piperonal afforded **64**, and lactone **65** was obtained by Super-Hydride-mediated ester reduction and concomitant lactonization. The geometry of the alkene was confirmed by analysis of the NOESY spectrum. With this substrate in hand, helioxanthin was obtained by benzannulation in 60% yield. The reaction mechanism can be explained as described below. Oxidative addition of Pd to aryl iodide **65** provided intermediate **66**, which is stabilized by a coordination of 1,3-diene to Pd even in the presence of steric hindrance generated by the aryl group of the diene. Then *syn* insertion of the palladium complex to the alkene provided σ-dihydronaphthalene palladium complex **67** and *syn* β-hydride elimination afforded helioxanthin. In the other process, the geometry of alkene can be isomerized to (*Z*) in **68** specified in red color in Scheme 6. In that case, the β-hydride, which is positioned *anti* to Pd in **69** is eliminated by *anti* β-hydride elimination process by way of π-allylpalladium complex **70**. In this synthesis, construction of the naphthalene ring is proceeded in relatively mild condition with only the catalytic amount of Pd catalyst in a concise, efficient manner with opposite regioselectivity usually obtained as a minor product using the Diels–Alder approach.

### Silver-Catalyzed Cyclization

**Scheme 13:**
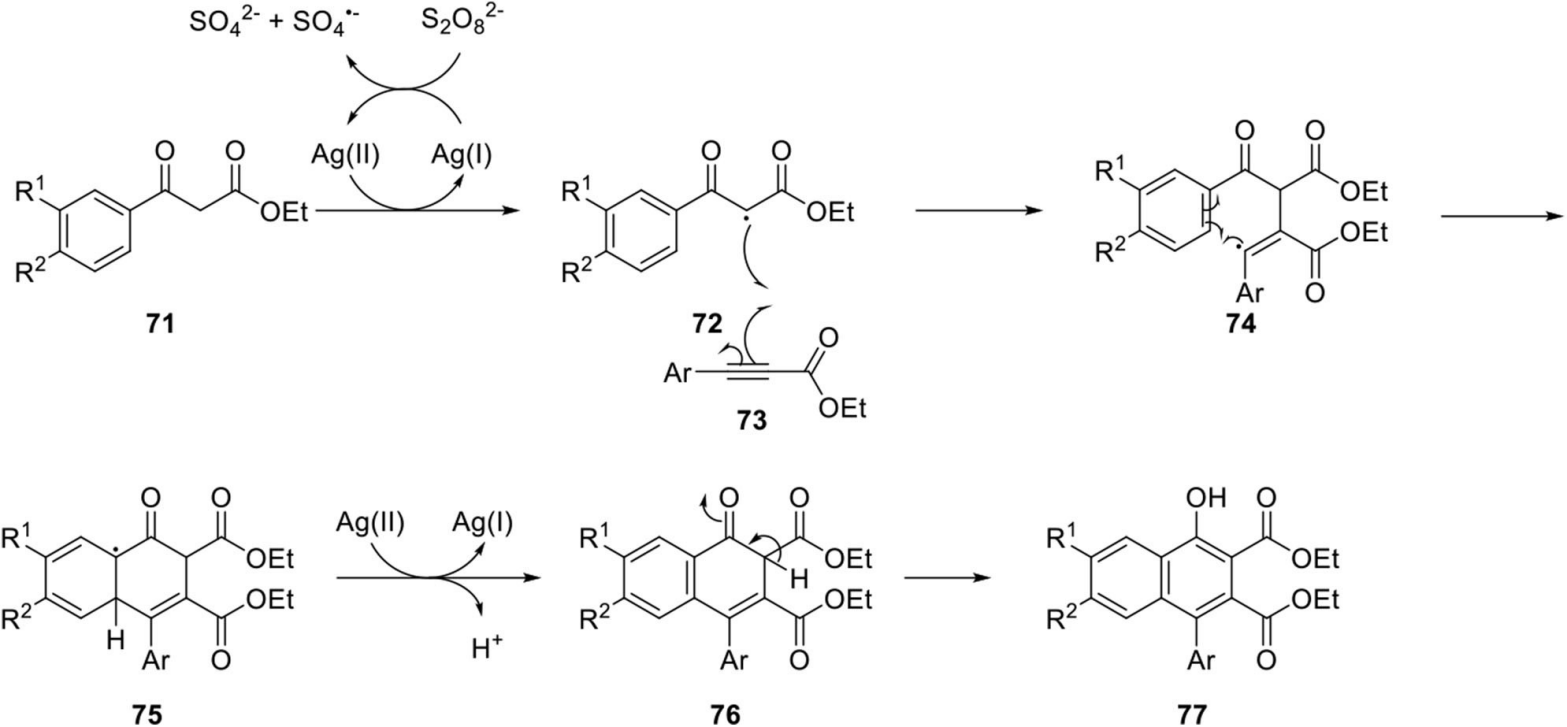
Mechanism of Ag-catalyzed cyclization of the β-keto ester.

Narender et al. developed a novel route for regioselective synthesis of 4-aryl substituted α-naphthols by silver(I) catalyzed C-H functionalization of β-keto esters **71** and aryl propiolates **73** in an environmentally friendly manner (Naresh et al., 2015). The reaction mechanism was proposed to begin with disproportionation of persulfate dianion to persulfate radical anion producing Ag(II) ion. Then, β-ketoester **71** is oxidized by the Ag(II) ion to a highly stable radical species **72**, and the radical is added to propiolate **73** to produce vinyl radical **74**, which is stabilized by the neighboring aryl group (Yan et al., 2015). Finally, the radical **74** underwent addition to the benzene ring and synthesis of 4-arylnaphthol **77** was achieved by additional oxidation of **75** by Ag(II) ion, and tautomerization of **76** (Scheme 13).

**Scheme 14:**
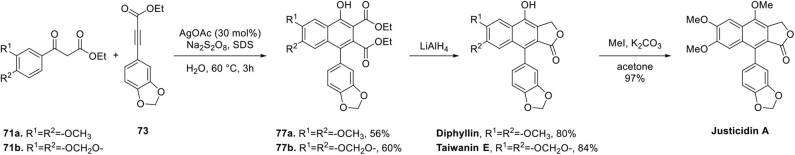
Synthesis of arylnaphthalene lignan lactone by Ag-catalyzed cyclization.

Successful and efficient applications for the synthesis of arylnaphthalene lignan lactone is summarized in Scheme 14. The reaction of substituted ethyl benzoyl acetate **71** and substituted ethyl phenylpropiolate **73** in the presence of silver acetate and Na_2_S_2_O_8_ furnished 4-aryl α-naphthol **77a** and **77b** in 56 and 60% yield, respectively. Surfactant (sodium dodecyl sulfate) was used to enhance water solubility and yield and reduced the use of organic solvent. Lithium Aluminum Hydride (LAH)-mediated reduction of ester located next to phenol and the spontaneous lactonization afforded diphyllin and taiwanin E in 80 and 84% yield, respectively. Justicidin A was also obtained by methylation of diphyllin in quantitative yield. This Ag-catalyzed cyclization proceeded in an atom economic manner, broad substrate scope, excellent regioselectivity, and environmental friendliness albeit with high loading of Ag catalyst.

## Conclusion

Arylnaphthalene lignan lactones continuously intrigue synthetic organic chemists and medicinal chemists owing to their structural uniqueness and pharmacological activity as well as the possibility as privileged structures. This review focused on transition metal catalyzed approaches for the synthesis of arylnaphthalene lignan lactones, especially on the construction of arylnaphthalene cores by metal catalyst incorporating gold, manganese, nickel, palladium, and silver. Compared with the non-metal catalyzed synthesis of arylnaphthalene lignan lactones, synthetic methodologies using transition metal catalysts provide distinct advantages in terms of mild reaction conditions, chemical yields, and functional group tolerance. In addition, single step construction of a complex core structure through multiple bond formation is scientifically meaningful and provides utility in chemical library synthesis for drug discovery.

## Author Contributions

DS conceptualized the manuscript. SP researched publications related to the review article. SP and J-HK prepared the schemes and figures. S-HK and DS wrote the draft of the manuscript and prepared the final version of the manuscript with considerable corrections. All authors approved the manuscript in its final form for publication.

## Conflict of Interest

The authors declare that the research was conducted in the absence of any commercial or financial relationships that could be construed as a potential conflict of interest.
